# Understanding the Natural History and the Effects of Current Therapeutic Strategies on Urea Cycle Disorders: Insights from the UCD Spanish Registry

**DOI:** 10.3390/nu17071173

**Published:** 2025-03-28

**Authors:** Elena Martín-Hernández, Marcello Bellusci, Patricia Pérez-Mohand, Patricia Correcher Medina, Javier Blasco-Alonso, Ana Morais-López, Javier de las Heras, Silvia María Meavilla Olivas, Lucy Dougherty-de Miguel, Maria Luz Couce, Elvira Cañedo Villarroya, María Concepción García Jiménez, Pedro Juan Moreno-Lozano, Inmaculada Vives, Mercedes Gil-Campos, Sinziana Stanescu, Leticia Ceberio-Hualde, María Camprodón, Elisenda Cortès-Saladelafont, Rafael López-Urdiales, Mercedes Murray Hurtado, Ana María Márquez Armenteros, Concha Sierra Córcoles, Luis Peña-Quintana, Mónica Ruiz-Pons, Carlos Alcalde, Fernando Castellanos-Pinedo, Elena Dios, Delia Barrio-Carreras, María Martín-Cazaña, Mónica García-Peris, José David Andrade, Camila García-Volpe, Mariela de los Santos, Angels García-Cazorla, Mireia del Toro, Ana Felipe-Rucián, María José Comino Monroy, Paula Sánchez-Pintos, Ana Matas, David Gil Ortega, Álvaro Martín-Rivada, Ana Bergua, Amaya Belanger-Quintana, Isidro Vitoria, Raquel Yahyaoui, Belén Pérez, Montserrat Morales-Conejo, Pilar Quijada-Fraile

**Affiliations:** 1 Unidad de Enfermedades Mitocondriales-Metabólicas Hereditarias, Hospital Universitario 12 de Octubre, Instituto de Investigación Imas12, MetabERN, CIBERER, 28041 Madrid, Spain; marcello.bellusci@salud.madrid.org (M.B.); patriciapilar.perez@salud.madrid.org (P.P.-M.); delia.barrio@salud.madrid.org (D.B.-C.); mmcazana@salud.madrid.org (M.M.-C.); montserrat.morales@salud.madrid.org (M.M.-C.); pilar.quijadaf@salud.madrid.org (P.Q.-F.); 2 Unidad de Nutrición y Metabolopatías, Hospital Universitario La Fé, 46026 Valencia, Spain; correcher_pat@gva.es (P.C.M.); garcia_monpera@gva.es (M.G.-P.); isivitoria@gmail.com (I.V.); 3 Unidad de Gastroenterología, Hepatología y Nutrición Pediátrica, Laboratorio de Bioquímica, Instituto de Investigación Biomédica de Málaga-IBIMA, Hospital Regional Universitario de Málaga, 29010 Málaga, Spain; javierblascoalonso@yahoo.es (J.B.-A.); raquelyahyaoui@gmail.com (R.Y.); 4 Unidad de Nutrición Infantil y Enfermedades Metabólicas, Hospital Universitario La Paz, 28046 Madrid, Spain; morais.lopez@salud.madrid.org (A.M.-L.); josedavid.andrade@salud.madrid.org (J.D.A.); anabergua@gmail.com (A.B.); 5 Hereditary Metabolic Diseases Unit, Hospital Universitario Cruces, MetabERN, Biobizkaia Health Research Institute, 48903 Bilbao, Spain; javier.delasherasmontero@osakidetza.eus (J.d.l.H.); letilu83@gmail.com (L.C.-H.); 6 Unidad de Enfermedades Metabólicas, Hospital San Joan de Déu, MetabERN, CIBERER, 08035 Barcelona, Spain; silviamaria.meavilla@sjd.es (S.M.M.O.); camila.garcia@sjd.es (C.G.-V.); marielamercedes.santos@sjd.es (M.d.l.S.); angeles.garcia@sjd.es (A.G.-C.); 7 Unidad de Enfermedades Metabólicas, Hospital Vall d’Hebrón, MetabERN, VHIR, 08035 Barcelona, Spain; lucydoughertydm@gmail.com (L.D.-d.M.); maria.camprodon@vallhebron.cat (M.C.); mireia.deltoro@vallhebron.cat (M.d.T.); ana.felipe@vllhebron.cat (A.F.-R.); 8 Unidad de Enfermedades Metabólicas, Hospital Clínico Universitario de Santiago, MetabERN, IDIS, 15706 Santiago de Compostela, Spain; maria.luz.couce.pico@sergas.es (M.L.C.); paula.sanchez.pintos@sergas.es (P.S.-P.); 9 Unidad de Nutrición y Enfermedades Metabólicas, Hospital Universitario Niño Jesús, 28009 Madrid, Spain; elviracaedo@yahoo.es; 10 Unidad de Metabolismo, Servicio de Pediatría, Hospital Miguel Servet, 50009 Zaragoza, Spain; igarciaji@salud.aragon.es; 11 Unidad de Errores Congénitos del Metabolismo en el Adulto, Medicina Interna, Hospital Clínic, 08036 Barcelona, Spain; pjmoreno@clinic.cat (P.J.M.-L.); anmatas@clinic.cat (A.M.); 12 Gastroenterología Pediátrica, Hospital Clínico Universitario Virgen de la Arrixaca, 30120 Murcia, Spain; vivesmas@hotmail.com (I.V.); d.gil.ortega@gmail.com (D.G.O.); 13 Unidad de Metabolismo, Hospital Universitario Reina Sofía, IMIBIC, UCO, 14004 Córdoba, Spain; mercedes_gil_campos@yahoo.es (M.G.-C.); maria.comino.monroy@gmail.com (M.J.C.M.); 14 Unidad de Enfermedades Metabólicas, Hospital Ramón y Cajal, MetabERN, 28034 Madrid, Spain; sinziana.stanescu@salud.madrid.org (S.S.); amaya.belanger@salud.madrid.org (A.B.-Q.); 15 Unidad de Neurología Pediátrica y Enfermedades Metabólicas, Hospital Germans Trias i Pujol, 08916 Barcelona, Spain; sendacs@gmail.com; 16 Departamento de Endocrinología y Nutrición, Hospital de Bellvitge, 08907 Barcelona, Spain; rafaellopez@bellvitgehospital.cat; 17 Unidad de Nutrición y Metabolopatías, Pediatría, Hospital Universitario de Canarias, 38320 Santa Cruz de Tenerife, Spain; mercedes.murray.hurtado@gmail.com (M.M.H.); amartinrivada@gmail.com (Á.M.-R.); 18 Gastroenterología Infantil, Hospital Materno-Infantil de Badajoz, 06010 Badajoz, Spain; anam.maar@gmail.com; 19 Neurología Pediátrica, Hospital Universitario de Jaén, 23007 Jaén, Spain; conchasierra@hotmail.com; 20 Gastroenterología y Nutrición Pediátrica, Complejo Hospitalario Universitario Insular-Materno Infantil, CIBEROBN-ISCIII, Universidad de Las Palmas de Gran Canaria, 35016 Las Palmas de Gran Canaria, Spain; lpena@dcc.ulpgc.es; 21 Unidad de Nutrición y Enfermedades Metabólicas, Pediatría, Hospital Universitario Virgen de la Candelaria, 38010 Tenerife, Spain; monicarpons@gmail.com; 22 Servicio de Pediatría, Hospital Universitario Río Ortega, 47012 Valladolid, Spain; calcalma@saludcastillayleon.es; 23 Neurología, Hospital Virgen del Puerto, Plasencia, 10600 Cáceres, Spain; fer.castellanosp@gmail.com; 24 Endocrinología y Enfermedades Metabólicas, MetabERN, Hospital Virgen del Rocío, 41013 Sevilla, Spain; elenadiosfuentes@gmail.com; 25 Centro de Diagnóstico de Enfermedades Moleculares, IdiPAZ, CIBERER, Universidad Autónoma Madrid, 28049 Madrid, Spain; bperez@cbm.csic.es

**Keywords:** urea cycle disorders (UCDs), arginase 1 (ARG1), argininosuccinate lyase (ASL), argininosuccinate synthetase (ASS1), carbonic anhydrase VA (CA-VA), citrin, carbamoylphosphate synthetase (CPS1), hyperornithinemia-hyperammonemia-homocitrullinuria (HHH), N-acetylglutamate synthase (NAGS), ornithine/citrulline antiporter (ORNT), ornithine transcarbamylase (OTC)

## Abstract

**Background/Objectives**: The present study updates the Spanish registry of patients with urea cycle disorders (UCD), originally established in 2013, to provide comprehensive epidemiological data and evaluate the impact of therapeutic strategies and newborn screening (NBS) on clinical outcomes. **Methods**: This retrospective, multicenter study focuses on 255 Spanish UCD patients. It includes all living and deceased cases up to February 2024, analyzing demographic, clinical, and biochemical variables. **Results**: The incidence of UCD in Spain over the past decade was 1:36,063 births. The most common defects were ornithine transcarbamylase deficiency (OTCD) and argininosuccinate synthetase deficiency. Early-onset (EO) cases comprised 32.7%, and 10.6% were diagnosed through NBS. Global mortality was 14.9%, higher in carbamoylphosphate synthetase 1 deficiency (36.8%) and male OTCD patients (32.1%) compared to other defects (*p* = 0.013). EO cases presented a higher mortality rate (35.8%) than late-onset (LO) cases (7.1%) (*p* < 0.0001). The median ammonia level in deceased patients was higher at 1058 µmol/L (IQR 410–1793) than in survivors at 294 µmol/L (IQR 71–494) (*p* < 0.0001). Diagnosis through NBS improved survival and reduced neurological impairment compared to symptomatic diagnosis. Neurological impairment occurred in 44% of patients, with worse neurological outcomes observed in patients with argininosuccinate lyase deficiency, arginase 1 deficiency, hyperornithinemia-hyperammonemia-homocitrullinuria, EO presentations, pre-2014 diagnosis, and patients with higher levels of ammonia at diagnosis. Among transplanted patients (20.6%), survival was 95.2%, with no significant neurological differences compared to non-transplanted patients. **Conclusions**: This updated analysis highlights the positive impact of NBS and advanced treatments on mortality and neurologic outcomes. Persistent neurological challenges underscore the need for further therapeutic strategies.

## 1. Introduction

Urea cycle disorders (UCDs) are a group of inherited metabolic diseases that impair nitrogen detoxification and the endogenous synthesis of arginine, citrulline, and ornithine [1]. The latest International Classification of Inherited Metabolic Disorders (ICIMD) [2] identifies 10 different diseases in the urea cycle and hyperammonemias group. This includes 5 enzymatic deficiencies directly affecting the urea cycle: carbamoyl-phosphate synthetase 1 deficiency (CPS1D), ornithine transcarbamylase deficiency (OTCD), argininosuccinate synthetase 1 deficiency (ASS1D-citrullinemia type 1), argininosuccinate lyase deficiency (ASLD-argininosuccinic aciduria), and arginase 1 deficiency (ARG1D). Additionally, there are two transporter defects—citrin deficiency (CITD) and mitochondrial ornithine/citrulline antiporter deficiency (ORNT1D), which causes hyperornithinemia-hyperammonemia-homocitrullinuria (HHH) syndrome. Two other enzyme deficiencies, carbonic anhydrase VA deficiency (CAVAD) and N-acetylglutamate synthase deficiency (NAGSD), impact the production of HCO3- and N-acetylglutamate, essential CPS1 substrates and cofactors, respectively. Finally, glutamate dehydrogenase superactivity (GLUD1) is also classified within this group. All these deficiencies are inherited in an autosomal recessive manner, except for OTCD and GLUD1 hyperactivity, which are inherited in an X-linked recessive and autosomal dominant manner, respectively [1].

The estimated cumulative incidence of UCDs is between 1:35,000 and 1:51,946 [3,4,5,6,7], with cases potentially manifesting at any age. Clinical presentation varies according to several factors, with the most significant being the severity of the deficiency and the affected gene. Early-onset (EO) or neonatal appear within 28 days of life and are typically associated with higher rates of mortality and morbidity, particularly due to neurological sequelae (motor and cognitive impairment) and a high risk of recurrence. In contrast, late-onset (LO) forms present after the neonatal period, exhibiting a more variable and generally less severe phenotype than EO cases [8,9,10].

Based on the stages of the urea cycle that are affected, UCDs can also be classified as proximal (NAGSD, CPS1D, and OTCD) or distal UCDs (ASS1D, ASLD, and ARG1D), where other metabolites accumulate in addition to ammonia (citrulline, arginine, and argininosuccinic acid) [1], permitting detection in the newborn screening.

Conventional treatment for UCDs consists of a protein-restricted diet, ammonia-scavenging drugs, and arginine/citrulline supplementation [1]. Although this approach has significantly improved patient survival, it does not stop decompensation episodes. Liver transplantation (LT) remains the only curative option, carrying procedure-related risks and requiring lifelong immunosuppression [8,11]. Consequently, alternative therapeutic strategies have been actively pursued in recent decades to enhance patient prognosis and quality of life. Currently, enzyme replacement therapy for arginase deficiency is available [12], and several investigational treatments, including adeno-associated viral (AAV) vector therapies, gene editing approaches, and mRNA-based therapies, are in development [13]. The combined impact of NBS for ASSD, ASLD, and ARG1D, alongside advances in nutritional and pharmacological therapies and LT, has contributed to a reduction in mortality rates (25% in EO and 11% in LO). However, despite these advances, neurodevelopmental outcomes remain suboptimal, particularly in the most severe cases [14,15,16,17,18].

In 2012, the Spanish Association of Inborn Errors of Metabolism (AECOM), with the aim of improving disease knowledge and care planning, initiated the establishment of a national registry of patients with UCDs, enrolling 104 patients [19]. The present study updates data on previously enrolled patients and includes those diagnosed after 2013, aiming to collect epidemiological information (prevalence, incidence, mortality) and assess long-term outcomes in the Spanish cohort. This updated analysis enables a comparison of our findings with those of recently published UCD registries from different countries [6,20,21,22,23] as well as with our earlier published data [19].

## 2. Materials and Methods

### 2.1. Study Design

This is an observational, retrospective, multicenter study involving patients with UCDs diagnosed in Spain, with both longitudinal and cross-sectional data collected from diagnosis until the last follow-up visit. The study was conducted by the UCD study group of the Spanish Association of Inborn Errors of Metabolism (AECOM). Data collection was conducted between March 2023 and February 2024. This encompassed all individuals diagnosed with UCD in Spain, regardless of age or survival status. Heterozygous females (mothers and sisters of patients with OTCD) were included only if they displayed symptoms or had biochemical abnormalities requiring medical treatment, while asymptomatic heterozygous females with OTCD were excluded. Study data were collected and managed using REDCap electronic data capture tools hosted at the Instituto de Investigación Hospital 12 de Octubre (imas12).

The study was approved by both the Local Ethics Committee of the Instituto de Investigación Hospital 12 de Octubre (imas12) (protocol Nº 22/629, 31 January 2023) and the Spanish authority. The study protocol was approved by the local research ethics committee of each center involved. Written informed consent was obtained from patients or their guardians to participate in the study.

### 2.2. Analyzed Variables

The data were retrospectively collected. At the time of diagnosis, the following information was recorded for each patient: sex, birth date, diagnosis date, disease (NAGSD, CPS1D, OTCD, ASS1D, ASLD, ARG1D, ORNT1D (HHH), CITD, and CAVAD), presentation (EO, LO, NBS, high-risk family screening (HRFS)), ammonia levels at presentation, and results from molecular studies. Between the date of diagnosis and the last visit, information on transplantation and mortality was also collected, including the date of transplantation and death. Additionally, the number of decompensation episodes with hyperammonemia exceeding 100 µmol/L during follow-up was documented. At the last visit, data recorded included each patient’s age, anthropometric data (weight, height, and head circumference), liver damage (hypertransaminasemia, liver hyper-echogenicity), neurological impairment (developmental delay, cognitive disability, learning disorder, behavioral disorders, motor disorders, epilepsy, and severe encephalopathy), children’s schooling and adults’ employment status and highest level of education, dietary treatment (total protein, essential amino acids, and supplements of L-arginine and/or L-citrulline), and pharmacological therapy.

Anthropometric parameters were collected following standard medical procedures at each center and converted into z-scores using the reference tables published by Carrascosa et al., which were based on Spanish population data [24].

The incidence for each type of UCD was calculated by dividing the number of diagnoses made between January 2014 and December 2023 by the annual number of live births during those years, as reported by the Spanish National Statistical Institute (INE) [25]. The results were expressed as the number of live births required to observe 1 case. Prevalence was determined by dividing the number of living patients by the Spanish population, based on INE data from December 2023 [25].

### 2.3. Statistical Analysis

Data are expressed as mean and standard deviation (SD) or median and interquartile range (IQR) for quantitative variables and percentages for qualitative variables. The level of statistical significance was obtained using Chi-square or Fisher’s Exact Test for qualitative variables and Student’s *t*-test or the Mann–Whitney U test for quantitative variables depending on the statistical distribution of the data. Spearman correlation was used for continuous variables. Statistical significance was established at *p* < 0.05. Survival curves were estimated using the Kaplan–Meier (product-limit) method. Analyses were performed using SAS software, Version 9.4 of the SAS System for Windows. Copyright © 2023 SAS Institute Inc., Cary, NC, USA.

## 3. Results

### 3.1. Patient Cohort Characteristics

This study included 255 patients from 24 centers across 11 regions in Spain; 104 of them were previously reported [19]. The distribution of diagnoses, detailed in Table 1, showed that the most common conditions were OTCD in 52.1% of cases and ASS1D in 21.2%. Within the OTCD group, 42.1% of patients were male. Symptom onset varied: 32.7% of cases had EO, 47.6% LO, and 19.7% were asymptomatic at diagnosis. Among the asymptomatic patients, 10.6% were identified through NBS and 9.0% through HRFS. The median age at diagnosis across the cohort was 9.96 months (IQR 0.24–45.24 months), with 76.0% of patients diagnosed by the age of 3 years. Symptomatic patients were diagnosed at the median age of 16.4 months (IQR 0.24–46.56 months), while asymptomatic patients (NBS or HRFS) were detected at a median age of 1.2 months (IQR 0.04–2.84 months). Most patients were of Spanish origin, representing 82.7% of the cohort, with smaller groups from other European countries, Morocco, and Latin America.

### 3.2. Epidemiological and Demographic Data

**Figure 1:**
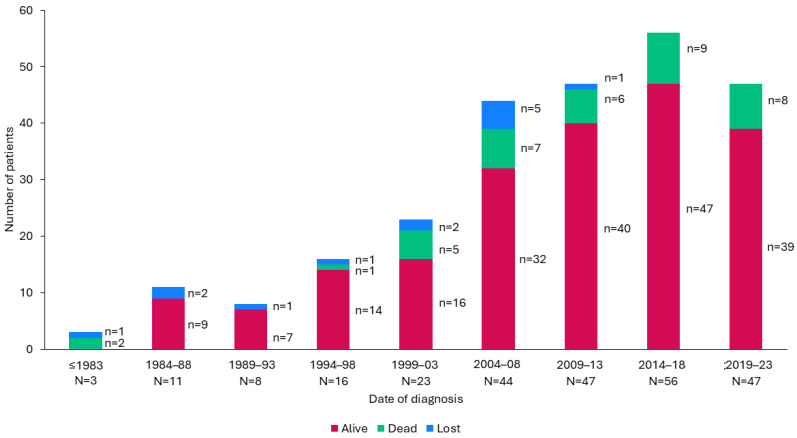
Number of cases according to date of diagnosis.

Of the 255 patients included, 103 were diagnosed between 2014 and 2023. The global incidence in the last 10 years was 1:36,063 births (95% CI, 29,719–44,164), and the prevalence at the end of 2023 was 1:238,200. The incidence for each disease is shown in Table 2. On average, there were 10.3 new cases annually and 1.7 deaths per year (Figure 1).

**Figure 2:**
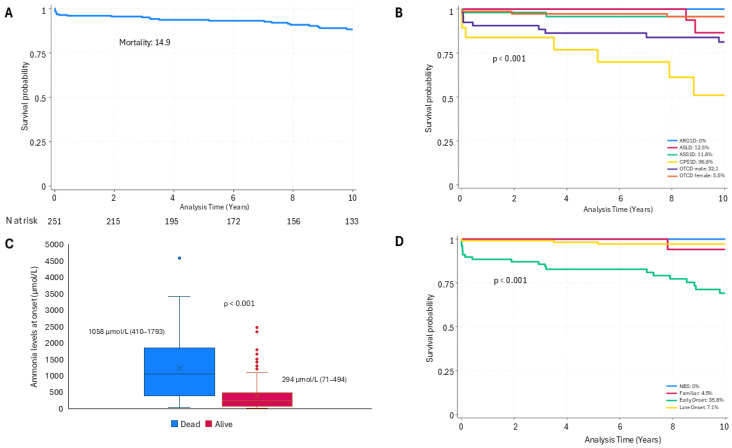
Mortality. (**A**) Overall survival estimate (Kaplan–Meier). (**B**) Survival by type of disorder (Kaplan–Meier). (**C**) Ammonia levels at onset. (**D**) Survival by clinical presentation time (Kaplan–Meier).

Mortality rates varied based on sex, diagnosis, onset type, and ammonia levels at presentation (Figure 2). Overall mortality for the cohort was 14.9% over a median follow-up of 10.6 years (IQR 4.1–17.8 years). The diagnosis of deceased patients included 21 with OTCD (55.3%), of whom 17 were males, 7 with CPS1D (18.4%), 6 with ASS1D (15.8%), 3 with ASLD (7.9%) and 1 with NAGSD (2.6%). None of the patients with another diagnosis died. The median age at death was 25.5 days (IQR 7.3 days–13 years), most deaths occurring within the first months of life. Among the 38 deceased patients, 65.8% were male and 34.2% female (*p* = 0.03). Mortality was higher in CPS1D cases (36.8%) and among male OTCD patients (32.1%) compared to other diagnoses (*p* = 0.013). Moreover, EO cases presented a higher mortality rate (35.8%) compared to those with LO (7.1%) (*p* < 0.0001). The median ammonia level in deceased patients was higher, 1058 µmol/L (IQR 410–1793 µmol/L), than in surviving patients, 294 µmol/L (IQR 71–494 µmol/L) (*p* < 0.0001). Excluding neonatal deaths, mortality was higher but not significantly in patients under medical management (MM) (6.3%) than in those who underwent LT (4.8%).

### 3.3. Follow-Up Outcomes

**Figure 3:**
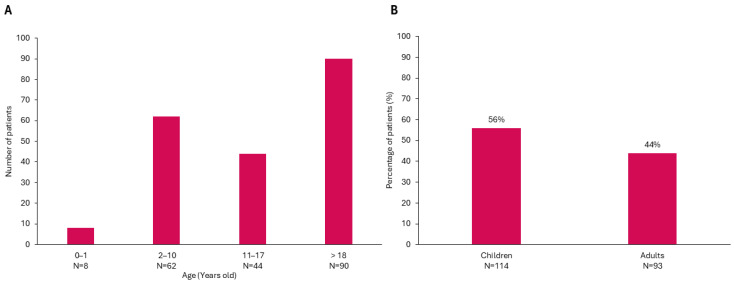
Age distribution of patients at follow-up. (**A**) Number of patients by age group. (**B**) Percentage of patients categorized as children. 109 females (53.4%) and 95 males (46.6%).

Over a median follow-up period of 10.6 years (IQR 4.1–17.8 years), 13 patients were lost to follow-up, 38 patients died (14.9%), and 204 remained under active follow-up. At the last follow-up, the median age was 16 years (IQR 8.20–26.37 years), with 44.1% of the patients now adults (Figure 3). The current distribution of diagnoses included 51.5% with OTCD (35% male), followed by 21.6% with ASS1D, 10.8% with ASLD, 5.9% with CPS1D, 4.4% with ARG1D, and smaller percentages for other UCDs. Symptom onset was EO in 25.5% of cases, LO in 51%, and 23.5% of patients remained asymptomatic at diagnosis. Of the asymptomatic cases, 13.2% were diagnosed by NBS, and 10.3% through HRFS.

#### 3.3.1. Profile of Patients Diagnosed Through NBS

A total of 27 patients were diagnosed by NBS, consisting of 18 with ASS1D, 7 with ASLD, and 2 with ARG1D. In ASS1D patients identified by NBS, the median age at last follow-up was 5.9 years (IQR 3.2–8.9 years). The median ammonia level at the time of the NBS confirmation was 60 µmol/L (IQR 48–109 µmol/L), with four patients presenting levels above 100 µmol/L. All but one of the ASS1D patients identified by NBS were on a protein-restricted diet, with nine receiving arginine supplements and four being treated with glycerol phenylbutyrate (GPB). All patients in this group were alive, and only one (5.5%) presented cognitive impairment. In contrast to ASS1D patients diagnosed through NBS, those diagnosed based on symptoms had a mortality rate of 17.0%; 56.0% exhibited cognitive impairment, 14% presented with epilepsy, and 15.6% received LT. Significant differences were observed in both mortality (*p* < 0.0001) and neurologic outcomes (*p* < 0.0004) when comparing ASS1D patients diagnosed by symptoms to those identified by NBS.

For ASLD patients diagnosed by NBS, the median age at last follow-up was 6.8 years (IQR 5.5–8.1 years). The median ammonia level was 68 µmol/L (IQR 53–147 µmol/L) at the confirmatory visit, with two patients having ammonia higher than 100 µmol/L. One patient was symptomatic on the day of the NBS confirmatory visit. This case was a severe case and led to liver transplantation at the age of 7 months. All ASLD patients identified by NBS were on a protein-restricted diet, with 5 receiving arginine supplements and 2 treated with GPB. All of them are alive, and 2 present cognitive impairment (28.5%), while 17.6% of ASLD patients diagnosed symptomatically had died. Among the survivors, all presented with neurological sequelae. Their median age at the last visit is 15.4 years (IQR 8.2–18.5 years) and five of them (38.5%) underwent transplantation. In summary, in the ASLD patient group, mortality (*p* < 0.001) and neurological outcomes (*p* < 0.001) were significantly higher in patients diagnosed by symptoms when compared to those identified by NBS.

The two ARG1D patients diagnosed by NBS were aged 15 and 6 years at the last follow-up and showed no neurological symptoms, unlike ARG1D patients diagnosed by symptoms or HRFS, who exhibited neurological impairment (*p* < 0.001). Of the symptomatically diagnosed patients, two of them have undergone LT.

#### 3.3.2. Anthropometric Data

The z-scores for weight, height, and head circumference were within the normal range for the Spanish population across all age groups; however, a subset of patients had values below −2SD (Table 3). Specifically, 7.9% had weight z-scores below −2SD, 14.1% had height z-scores below −2SD, and 8.3% had head circumference z-scores below −2SD. Height Z-scores tended to decrease with age and were significantly associated with the prescribed proteins (*p* < 0.001).

#### 3.3.3. Neurological Outcome

Neurological or neuropsychological impairment was noted in 43.8% of the patients under follow-up, primarily with cognitive rather than motor dysfunction (Table 4). A subset of 46 patients underwent detailed neuropsychological assessment, with a median intelligence quotient of 87. Movement disorders were seen in 13.0% of patients, and 7.3% required wheelchair assistance. School outcomes indicated that 58.3% of children attended regular education, 25.0% received additional support, 13.0% were in special education, and 3.7% were not yet of school age but displayed normal neurodevelopmental outcomes. Among adults, 48.5% were employed, 2.9% were in sheltered employment, and 14.7% were studying. Living arrangements showed that 42.4% of adults lived independently, 8.2% in supported housing, and 49.3% with parents.

Neurological outcomes were worse in patients with ASLD, ARG1D, and HHH (*p* = 0.02), in EO patients (*p* < 0.0001), and in those with higher levels of ammonia at diagnosis (*p* < 0.001). The mean ammonia value was related to neurological outcomes in OTCD (*p* = 0.006) and ASS1D patients (*p* = 0.01). The incidence of neurologic sequelae has dropped from 49.6% in those diagnosed before 2014 to 35.7% in those diagnosed in the last 10 years (*p* = 0.05). However, no significant difference was observed between transplanted or non- transplanted patients (*p* = 0.36) (Table 4).

#### 3.3.4. Liver Disease

Excluding LT patients, 20.1% of the cohort presented liver disease at the last follow-up, characterized by persistent or intermittent hypertransaminasemia or liver hyper-echogenicity. Liver disease was most prevalent among patients with HHH (60%), ARG1D (43%), ASLD (25%) and OTCD (20%).

#### 3.3.5. Treatment

##### Medical Treatment

Of the 164 patients on MM, 92.0% followed a protein-restricted diet, and 43.7% received essential amino acid supplements, contributing to 17.5–30% of total protein intake. Table 3 shows the protein prescribed and the essential amino acid supplements by age groups. Prescribed protein intake decreased with age and was related to height (*p* < 0.001).

Additionally, Table 5 summarizes the pharmacological treatments and supplementation regimens. Citrulline and/or arginine supplements were provided to 76.2% of patients on medical management: citrulline alone to 53 patients (46 OTCD, 5 CPS1D, 2 HHH), arginine alone to 51 patients (27 ASS1D, 15 ASLD, 7 OTCD, 1 NAGSD, 1 HHH); and both to 21 patients (15 OTCD, 4 CPS1D, 1 NAGSD, 1 HHH). Furthermore, 14 transplanted patients (35%) received treatment with citrulline or arginine. The doses are shown in Table 5. The dose of arginine in distal UCDs (107 ± 67 mg/kg/day) was similar to that in proximal UCDs and HHH (101± 63 mg/kg/day) among the patients diagnosed in the last ten years.

Ammonia scavenger therapy was also used by 60.0% of the cohort (70% of symptomatic patients), mainly GPB (81.6%), with smaller groups receiving NaBZ, NaPB, or combined therapies. Patients on GPB were younger (mean age of 17.3 ± 12.2 years) than those on NaBZ or NaPB (mean ages of 29.6 ± 9.3 and 27.1 ± 11.6 years, respectively). Three patients received treatment with carglumic acid: two were diagnosed with NAGSD and the other with CAVAD. The latter also received treatment with GPB and presented a severe form of the disease, likely due to the presence of two mutations in the CAVA gene in addition to a mutation in the CPS1 gene.

##### Transplanted Patients

Among 42 transplanted patients, 40 were alive with a survival rate of 95.3%. The median follow-up period post-transplant was 6.1 years (IQR 2.5–11.7 years). Of the two deceased patients, one was a female with OTC deficiency who underwent transplantation at 2.9 years of age and died from chronic rejection 26 years post-transplant. The other was a male who underwent transplantation for liver failure at 15 months of age and died one week post-transplant; he was diagnosed post-mortem with OTC deficiency. The median age of the transplanted patients at follow-up was 13.6 years (IQR 8.5–19.3 years), and the median age at transplantation was 5.6 years (IQR 2.4–9.7 years), with 45.0% male. The most predominant diagnoses were OTCD (59.5%), followed by ASS1D (16.6%), ASLD (14.4%), CPS1D (4.8%), and ARG1D (4.8%). Regarding disease onset, 47.6% had neonatal onset, 45.2% had late onset, 4.8% were diagnosed through family screening, and 2.8% were diagnosed through neonatal screening (ASLD). In the transplanted patient group, 35.0% required supplementation with citrulline or arginine.

As shown in Table 6, which compares outcomes between patients managed with LT and MM, the LT group includes patients with more severe clinical presentations. This is reflected by the significantly higher percentage of EO cases (50.0% vs. 19.5%, *p* < 0.001), elevated ammonia levels at onset (507 ± 451 μmol/L vs. 342 ± 425 μmol/L, *p* = 0.04), and a greater number of HAEs per year (1.57 ± 2.99 vs. 0.37 ± 1.94, *p* = 0.0196). Despite this, the LT group had a lower mortality rate, although not statistically significant and achieved a complete resolution of HAEs during follow-up (0 vs. 0.16 ± 0.45 episodes/year, *p* < 0.001). Growth and neurological involvement were similar in both groups, with no significant differences.

## 4. Discussion

To summarize the principal findings of the present study:

1. Epidemiologic data revealed a cumulative incidence over the past decade of approximately 1 in 36,063 live births, a prevalence of 1 in 238,200 by the end of 2023, and a 14.9% mortality rate with a median follow-up of 10.6 years.
2. Mortality rates varied by UCD subtype, being higher in patients with CPS1D and in males with OTCD. Neonatal onset and peak ammonia levels at diagnosis were also linked to increased mortality. NBS-diagnosed patients had a favorable short-term prognosis; however, long-term outcomes need to be reassessed over time.
3. Neurological disability was observed in 44.0% of patients, especially among those with ASLD, ARG1D, and HHH. Notably, neurological impairment was reduced to 36.0% in patients diagnosed within the past decade, compared to 50.0% in earlier cases.
4. Liver transplantation was performed in 18.0% of those surviving the neonatal period, yielding excellent survival rates. However, neurological outcomes and growth in transplanted patients were similar to those medically managed.

This study provided an update on the Spanish UCD registry, providing insights into the epidemiological characteristics and clinical outcomes. Over the past decade, the cumulative incidence has been approximately 1 in 36,063 live births, with 9–10 new cases diagnosed annually, similar to rates in the U.S. (1:35,000) [5] and Finland (1:39,000) [4] and higher than rates in Italy (1:41,506) [3], Japan (1:50,000) [7], and the Germany-Switzerland-Austria region (1:52,000) [6]. However, differences in inclusion criteria and patient demographics across studies, such as restricting participants to those under 16–17 years of age or to living patients, may lead to underestimations of incidence in certain regions. In this context, long-term follow-up revealed an increase in median patient age, with 44.0% now over 18, up from 25.0% a decade ago, highlighting the role of adult metabolic units in ensuring continuity of care in Spain. This finding underscores the importance of including adult patients in incidence estimation. Additionally, our inclusion of deceased patients helps avoid underreporting early deaths in severe cases, as seen in registries that only include living individuals [22].

Published data highlight geographic variations in UCD subtype distribution. Regional differences include a higher prevalence of ASLD in Finland [4], CPS1D accounting for 16.0% of cases in Central Europe [6], and arginase deficiency being the second most frequent disorder in Portugal [28]. Despite these regional variations, OTCD is consistently the most common disorder worldwide, typically followed by ASS1D and ASLD, which together comprise 30.0% of cases, while other subtypes each occur at frequencies below 5.0% [5,14]. These findings highlight the need for a country-specific analysis to better understand the regional landscape of the disease and adapt healthcare systems to the specific needs of each patient population.

In this context, our study analyzed mortality rates within the cohort, revealing a global rate of 14.9%. Mortality was higher in EO patients (35.8%) compared to LO cases (7.1%), with no deaths among NBS-diagnosed cases, consistent with previous studies [6,29]. Italian studies report a slightly higher mortality rate (23.1%), likely due to limited treatment availability prior to 1997 [3]. In contrast, Japanese data linked improved survival in male OTCD patients to early LT (performed in 23.7% of cases) [23], while UCDC (Urea Cycle Disorders Consortium) and E-IMD (European Registry and Network for Intoxication Type Metabolic Diseases) registries report lower rates (4.9%), likely because they track mainly surviving patients [22]. Similar to the Central European study [6], our cohort’s mortality was linked to EO presentations, CPS1D, male OTCD, and high ammonia at diagnosis. These data emphasize the importance of early diagnosis, expanded NBS, and effective ammonia control to improve UCD survival and outcomes.

NBS has proven to be an essential tool for early UCD detection; however, NBS implementation varies widely across regions. For instance, while the U.S. universally applies NBS for ASS1D and ASLD, European countries have been slower to adopt this approach due to concerns about the efficacy and outcomes of early treatment [1,30]. In Spain, NBS for distal UCDs, including ASS1D, ASLD, and ARG1D, has only been implemented in certain regions. As a result, 10.6% of patients in our series (13% of patients at follow-up) were diagnosed through NBS, an increase from 4.8% in 2014 [19]. This rise in NBS diagnosed cases aligns with the rates reported in the UCDC and E-IMD registries (8.9%) [20,21,22] but remains lower than the 20% reported in the Central European study [6]. Such progress indicates improvement in early detection, although a more universal approach in Spain and across Europe could further enhance outcomes for UCD patients.

NBS-identified patients in our cohort showed significantly better short-term outcomes than those diagnosed later, highlighting the impact of early detection. However, screening often captures milder phenotypes, complicating management and treatment intensity decisions [31]. In our cohort, no NBS-diagnosed patients with ASS1D or ASLD have died, with neurological impairment rates at 5.5% and 28.5%, respectively, compared to 56.0% and 100.0% in symptom-diagnosed patients. Previous studies found that NBS frequently identifies attenuated phenotypes with higher residual enzymatic activity [32,33], explaining milder clinical presentations. However, ASLD patients still exhibit high cognitive impairment despite early detection by NBS or HRFS [34,35,36], suggesting additional factors beyond hyperammonemia may influence clinical outcomes [35,36].

For ARG1D, early detection through NBS is crucial due to its subtle presentation, which often delays diagnosis and leads to irreversible neurological complications [37]. In our study, neither of the two NBS-diagnosed ARG1D patients showed neurological issues, while all symptomatically diagnosed patients had deficits. This contrast underscores the role of NBS in improving outcomes, especially with the recent approval of enzyme replacement therapy for ARG1D [12]. Early diagnosis thus appeared essential not only for immediate treatment but also to prevent the gradual, progressive neurological damage associated with this disorder.

Although the benefits of NBS are clear, predicting the severity in distal UCDs identified through screening remains challenging, which affects the planned intensity of treatment [31]. In our cohort, treatment strategies were informed by clinical, biochemical, and genetic data, consistent with recommendations published by Italian experts [38].

Anthropometric data underscores the positive impact of improved management. While most patients reached normal weight and height, adult patients in our cohort had a lower height Z-score, possibly due to dietary restrictions during growth. Height correlated with prescribed dietary protein, differing from studies attributing growth issues in symptomatic patients to disease severity and low branched-chain amino acid (BCAA) concentrations rather than protein restriction alone [39]. Further findings show positive links between height, L-leucine, L-valine, and dietary protein-to-energy ratios [40,41], emphasizing the need for individualized nutrition to support healthy growth in UCD patients.

Cognitive and motor deficits remain common in individuals with inherited UCDs, affecting 44.0% of the cohort despite advancements in treatment. However, prognosis has improved significantly over the past decade, with disability rates declining to 36.0% from 50.0% in previous years, highlighting advancements in diagnosis and treatment (Table 4). As previous studies show [15,16,17,18,42,43], poorer outcomes are linked to EO cases, ASLD, ARG1D, and HHH syndrome, with high ammonia levels at diagnosis correlating with impairment, especially in proximal UCDs [8,23,42]. Supporting this, ammonia levels above 300–360 µmol/L have been associated with impaired neurodevelopment [23], highlighting the need for early and aggressive treatment to mitigate neurological outcomes. However, ammonia levels were not significant predictors of neurological outcomes in ASLD and ARG1D cases, where cumulative exposure to specific biomarkers [14,16,44] or alternative mechanisms, such as disruptions in the nitric oxide pathway and oxidative stress, may play a contributory role [35,36,44,45]. In our cohort, 75.0% of patients without neurological impairment had ammonia levels below 316 µmol/L, further emphasizing the benefits of early ammonia control. Notably, neurological outcomes did not significantly differ between transplanted and non-transplanted patients as reported previously [11,23,46]. This suggests that while LT supports metabolic stability, it may not substantially alter neurodevelopmental outcomes.

Beyond early diagnosis, long-term UCD treatment strategies have evolved, focusing on dietary and pharmacological approaches and emergency protocols for hyperammonemia events [1,47]. In our cohort, 92.0% of MM patients followed a protein-restricted diet, with 44.0% receiving essential amino acids (EAAs). This approach aligns with WHO recommendations [26] and European practices [27,38,48]. In the U.S., EAAs are recommended for all patients, providing 50% of total protein intake during infancy, with this percentage later reduced to 25% [49]. EAAs help reduce the ammonia load in the urea cycle, and their higher BCAA content compensates for deficiencies in patients treated with phenylbutyrate.

Citrulline and arginine supplementation remain key in UCD management, with 76.0% of patients in our cohort receiving guideline-aligned doses [1]. Evidence now supports lower arginine doses in distal UCDs, particularly ASLD, to limit argininosuccinic acid buildup [50]. In our study, most patients with OTCD, CPS1D, and HHH syndrome were treated with L-citrulline. Traditionally, L-citrulline was preferred in OTC and CPS deficiencies because it has the advantage of incorporating aspartate into the pathway and removing an additional nitrogen molecule. However, it remains unclear whether L-citrulline, L-arginine, or a combination of both is more effective. Recent evidence suggests that L-citrulline is more effective in treating proximal UCDs, leading to higher plasma arginine levels compared to L-arginine [38,51,52].

In addition to dietary management, nitrogen scavengers are essential for ammonia reduction. In this cohort, 71.0% of symptomatic patients (60.0% overall) received nitrogen scavengers, primarily GPB, available in Spain since 2018. Since its introduction, 48 patients switched from NaPB or NaBZ to GPB, achieving better metabolic control and fewer adverse effects [53]. UK studies confirm the benefits of GPB treatment, showing reduced propylene glycol and sodium exposure, with better patient acceptance due to lower volume and improved taste [54,55]. Recently, a new NaPBA formulation has been developed, potentially allowing for lower doses when taken while fasting [56].

LT remains essential for cases where MM fails to prevent hyperammonemic decompensation [1]. Balancing the risks of LT is particularly challenging in mildly to moderately affected patients, making treatment decisions difficult for both families and professionals. In Spain, LT rates in UCD patients rose from 5% in 2014 to 19.6% of patients at follow-up in 2024, exceeding the 10% in UCDC and E-IMD registries [22], but lower than Japan, where early LT has notably improved survival, particularly in male OTCD patients [23]. In our cohort, LT outcomes have been positive, with a 95% survival rate, prevention of hyperammonemic crises, and increased dietary flexibility. However, growth and neurological outcomes in transplanted patients were similar to those medically managed, likely due to greater disease severity in LT patients. Nevertheless, in a recent study of patients from the UCDC and E-IMD registries (OTCD, ASS1D, and ASLD), stratified by severity, those who underwent LT were compared with those on medical management. The LT group demonstrated improved metabolic stability and was associated with more favorable growth outcomes [39].

Other studies indicate that while LT provides metabolic stability, it does not significantly improve neurodevelopmental outcomes compared to MM [11,23], though it may prevent progressive impairment in argininemia cases [23,28]. Additionally, recent research from the Patient-Centered Outcome Research Institute in the U.S. found that, despite metabolic benefits, LT showed no statistically significant differences in mortality, quality of life, or neuropsychological function compared to MM alone [57]. This highlights the need for further therapeutic advances in UCD treatment and the need for further studies to assess long-term outcomes.

### Strengths and Weaknesses

This multicenter study provides comprehensive epidemiological data on UCDs in Spain, enabling robust analysis of clinical practices and outcomes. The large, diverse patient cohort enhances generalizability, and the inclusion of both living and deceased patients offers a complete picture of disease burden, minimizing underreporting of early mortality. Increased NBS implementation across regions allows assessment of early diagnosis benefits to guide future strategies. Similarly, the inclusion of patients of all ages, particularly the significant proportion of adults, highlights the increasing number of adult patients, who are likely to have unmet needs that will require attention (e.g., limited employment opportunities, many still living with their parents). However, as a retrospective study, it may be subject to biases in data collection, and variability in treatment protocols across centers could affect outcome comparability. Despite these limitations, this study provides valuable insights into UCD epidemiology and management in Spain, laying a foundation for future research and improvements in patient care.

## 5. Conclusions

This updated analysis of the Spanish UCD registry highlights advances in diagnosis and management. The broader implementation of NBS has doubled detection rates since 2014, with NBS-diagnosed patients showing fewer neurological complications. Long-term UCD management relies on dietary adjustments, amino acid supplementation, and nitrogen scavengers to support metabolic stability. Although most patients reach normal growth parameters, lower adult height Z-scores may reflect early dietary limitations. LT remains essential for patients who are unresponsive to medical treatment, though it has limited neurodevelopmental impact. The Spanish cohort data provide a strong foundation for further research, with upcoming analyses exploring phenotype-genotype correlations in NBS-detected patients to predict severity and personalize treatment. Over time, these data will be crucial for assessing new therapies’ impact on mortality, neurological outcomes, and quality of life, advancing UCD patient care.

## Figures and Tables

**Table 1:** Characteristics of the series.

Disease	Patients *N* = 255	Gender *N* = 255		Presentation *N* = 254			Evolution *N* = 255			Treatment in Patients Under Follow-Up *N* = 204	
		Male	Female	Symptomatic		Asymptomatic	Alive	Died	Lost	MM	LT
				EO	LO						
OTCD, N (%)	133 (52.1)	-	-	27 (20.3)	88 (66.2)	17 (12.8)	105 (78.9)	21 (15.8)	7 (5.3)	82 (68.7)	23 (17.3)
*OTCD-M* *(%)*	-	56 (42.1)	-	23 (41.1)	26 (46.4)	6 (10.7)	36 (64.3)	17 (30.3)	3 (5.4)	28 (77.8)	8 (22.2)
*OTCD-F* *(%)*	-	-	77 (57.9)	4 (5.2)	62 (80.5)	11 (14.3)	69 (89.6)	4 (5.2)	4 (5.2)	54 (78.3)	15 (21.7)
ASS1D, N (%)	54 (21.2)	36 (66.6)	18 (33.3)	27 (50.0)	8 (14.8)	19 (35.2)	44 (81.5)	6 (11.1)	4 (7.4)	37 (84.1)	7 (15.9)
ASLD, N (%)	26 (10.2)	11 (42.3)	15 (57.7)	11 (42.3)	6 (23.1)	9 (34.6)	22 (84.6)	3 (11.5)	1 (3.8)	16 (72.7)	6 (27.3)
CPS1D, N (%)	19 (7.4)	9 (47.4)	10 (52.6)	11 (57.9)	7 (36.8)	1 (5.3)	12 (63.1)	7 (36.8)	0 (0)	10 (83.3)	2 (16.7)
ARG1D, N (%)	9 (3.5)	4 (44.4)	5 (55.5)	1 (11.1)	5 (55.5)	3 (33.3)	9 (100)	0 (0)	0 (0)	7 (77.8)	2 (22.2)
NAGSD, N (%)	4 (1.6)	2 (50.0)	2 (50.0)	3 (50.0)	1 (50.0)	0 (0)	2 (50)	1 (25.0)	1 (25.0)	2 (100)	0 (0)
ORNT1D, N (%)	5 (2.0)	4 (80.0)	1 (20.0)	2 (40.0)	3 (60.0)	0 (0)	5 (100)	0 (0)	0 (0)	5 (100)	0 (0)
CITD, N (%)	3 (1.2)	3 (100)	0 (0)	1 (33.3)	1 (33.3)	1 (33.3)	3 (100)	0 (0)	0 (0)	3 (100)	0 (0)
CAVAD, N (%)	2 (0.8)	1 (50.0)	1 (50.0)	0 (0)	2 (100)	0 (0)	2 (100)	0 (0)	0 (0)	2 (100)	0 (0)
TOTAL, N (%)	255 (100)	126 (49.4)	129 (50.6)	83 (32.7)	121 (47.6)	50 (19.7)	204 (80.0)	38 (14.9)	13 (5.1)	164 (80.4)	40 (19.6)

Abbreviations: ARG1D: arginase 1 deficiency; ASLD: argininosuccinate lyase deficiency; ASS1D: argininosuccinate synthetase deficiency; CAVAD: carbonic anhydrase VA deficiency; CITD: Citrin mitochondrial aspartate/glutamate carrier deficiency; CPS1D: carbamoyl-phosphate synthetase 1 deficiency; LT: liver transplantation; MM: medical management; NAGSD: N-acetylglutamate synthase deficiency; ORNT1D: ornithine/citrulline antiporter deficiency, HHH syndrome; OTCD: ornithine transcarbamylase deficiency; OTCD-F: OTC female; OTCD-M: OTC male.

**Table 2:** Incidence of urea cycle disorders.

Disease	Total Number of Cases, *n* (%)	Number of Cases Diagnosed Between 2014 and 2023, *n*	Incidence in 2014 and 2023 *	Mortality, *n* (%)
NAGSD	4 (1.6)	3	1:1,238,172	1 (25.0)
CPS1D	19 (7.4)	13	1:285,732	7 (36.8)
OTCD	133 (52.1)	33	1:112,561	21 (15.8)
OTCD males	56 (42.1)	16	1:232,002	17 (30.3)
OTCD females	77 (57.9)	17	1:218,355	4 (5.2)
ASS1D	54 (21.2)	26	1:142,770	6 (11.1)
ASLD	26 (10.2)	14	1:265,146	3 (11.5)
ARG1D	9 (3.5)	7	1:530,645	0
ORNT1D (HHH)	5 (2.0)	3	1:1,238,172	0
CITD	3 (1.2)	2	1:1,857,258	0
CAVAD	2 (0.8)	2	1:1,857,258	0
TOTAL	255 (100)	103	1:36,063	38 (14.9)

Abbreviations: ARG1D: arginase 1 deficiency; ASLD: argininosuccinate lyase deficiency; ASS1D: argininosuccinate synthetase deficiency; CAVAD: carbonic anhydrase VA deficiency; CITD: Citrin deficiency. CPS1D carbamoylphosphate synthetase 1 deficiency; NAGSD: N-acetylglutamate synthase deficiency; ORNT1D (HHH): ornithine/citrulline antiporter deficiency; OTCD: ornithine transcarbamylase deficiency. * Incidence for each type of urea cycle disorder was calculated by dividing the number of diagnoses made between January 2014 and December 2023 by the annual numbers of live births for those years 3,712,044 [25].

**Table 3:** Anthropometric and dietetic data according to age group.

	0 ≤ 2 Years (*N* = 8)		2–10 Years (*N* = 62)	11–18 Years (*N* = 44)	>18 Years (*N* = 90)	Global (*N* = 204)
Age Median years (IQR)	1.38 (1.18–1.51)		5.91 (4.19–8.75)	14.61 (12.92–16.54)	28.97 (21.46–38.33)	16.51 (8.23–26.60)
Weight Median kg (IQR) Z-score *	10.09 (8.08–11.35) −0.47		21 (15.43–27) −0.05	44.45 (37.48–50) −0.87	61.15 (55.45–72.83) 0.19	45.85 (24.68–60.78) −0.13
Height Median cm (IQR) Z-score *	79.50 (68.25–82.45) −0.85		111 (100.8–125) −0.50	153.50 (145.38–161.38) −0.87	164.00 (156.10–170) −0.9	151.80 (120.13–163) −0.77
HC Median cm (IQR) Z-score *	48.41 (46.6–49.1) −0.35		51 (49.07–51.95) −0.25	54.00 (53.86–54.72) 0.21	55.11 (54.78–55,81) −0.35	52.00 (49.65–54.00) 0.02
Total proteins Median g/kg/d (IQR)	1.55 (1.43–1.90)		1.20 (0.9–1.49)	0.80 (0.58–1.00)	0.76 (0.63–0.90)	0.88 (0.70–1.20)
EAs Median (g/kg/d) (IQR) (%)	0.27 (0.24–0.44) (17.4%)		0.29 (0.2–0.37) (24.0%)	0.16 (0.11–0.31) (20.0%)	0.23 (0.11–0.36) (30.0%)	0.25 (0.15–0.37) (28.4%)
Age	**<6 m**	**6–12 m**	**1–10 years**	**11–16 years**	**>16 years**	-
WHO/FAO/UNU **	1.77	1.31	0.92–1.14	0.84–0.90	0.84–0.87	-
UK (*N* = 45) ***	2 (0.7–2.5)	1.6 (1.2–1.8)	1.3 (1–1.7)	0.9 (0.7–1.4)	0.8 (0.4–1.2)	-

Abbreviations: HC: head circumference; EAs: essential amino acids. * Z-score from tables by Carrascosa et al. [24]. ** WHO/FAO/UNU Expert Consultation: Protein and Amino Acid Requirements in Human Nutrition [26]. *** British Inherited Metabolic Diseases Group (BIMDG) Dietitian’s Group [27].

**Table 4:** Clinical and biochemical data in relation to neurological outcome.

	*n*	Neurologically Impaired, *n* (%)	Not Neurologically Impaired, *n* (%)	*p*-Value
Total	201	88 (43.8)	113 (56.2)	
Disease				0.02
OTCD male	35	13 (37.1)	22 (62.8)	
OTCD female	68	23 (33.8)	45 (66.2)	
ASS1D	43	16 (37.2)	27 (62.8)	
ASLD	22	15 (68.2)	7 (31.8)	
CPS1D	12	7 (58.3)	5 (41.6)	
ARG1D	9	7 (77.7)	2 (22.2)	
NAGSD	2	1 (50.0)	1 (50.0)	
ORNT1D (HHH)	5	4 (80.0)	1 (20.0)	
CITD	3	1 (33.3)	2 (66.6)	
CAVAD	2	1 (50.0)	1 (50.0)	
Onset				<0.0001
Neonatal	52	37 (71.1)	15 (28.8)	
Late	101	45 (44.5)	56 (55.4)	
Asymptomatic	48	6 (12.5)	42 (87.5)	
Year of diagnosis				0.05
2014–2023	84	30 (35.7)	54 (64.3)	
<2014	117	58 (49.6)	59 (50.4)	
Laboratory test at onset				<0.001
Ammonia (µmol/L)	92	376 (80–800)	201 (60–316)	
Treatment				NS
Transplanted	39	20 (51.3)	19 (48.7)	
Non-transplanted	162	68 (42.0)	94 (58.0)	

Abbreviations: ARG1D: arginase 1 deficiency; ASLD: argininosuccinate lyase deficiency; ASS1D: argininosuccinate synthetase deficiency; CAVAD: carbonic anhydrase VA deficiency; CITD: citrin deficiency. CPS1D carbamoylphosphate synthetase 1 deficiency; NAGSD: N-acetylglutamate synthase deficiency; NS: non-significant; ORNT1D (HHH): ornithine/citrulline antiporter deficiency; OTCD: ornithine transcarbamylase deficiency.

**Table 5:** Pharmacological treatment and supplements of arginine and citrulline.

Drug	Patients, *n*	Mean ± SD (mg/kg/day)
NaPB	7	209 ± 121
NaBZ	11	202 ± 3
GPB	86	233 ± 94
Carglumic acid	3	75 ± 66
Arginine	72	148 ± 113
Citrulline	74	159 ± 83

Abbreviations: GPB: glycerol phenylbutyrate; NaBZ: sodium benzoate; NaPB: sodium phenylbutyrate; SD: standard deviation. 125 patients received arginine and/or citrulline, and 98 patients received scavengers. 21 patients received both citrulline and arginine, and 7 patients received combined scavengers.

**Table 6:** Health outcomes in patients with liver transplantation and medical management.

Patients at Follow-Up *N* = 204	LT *N* = 40 (19.6%)	MM *N* = 164 (80.4%)	*p*-Value
Weight (mean z-score ± SD)	−0.83 ± 1.11	0.09 ± 1.41	<0.001
Height (mean z-score ± SD)	−0.80 ± 0.24	−0.84 ± 1.34	NS
HC (mean z-score ± SD)	−0.66 ± 0.96	−0.3 ± 1.45	NS
Neurological involvement (%)	51.3%	41.4%	NS
HAEs/patient/year * (mean ± SD)	1.57 ± 2.99	0.37 ± 1.94	0.019
HAEs/year in the last two years (mean ± SD)	0	0.16 ± 0.45	<0.001
EO (%)	(50.0%)	(19.5%)	<0.001
Ammonia at onset (mean ± SD) (µmol/L)	507 ± 451	342 ± 425	0.040

Abbreviations: EO: early onset; HAEs: hyperammonemia crisis with ammonia > 100 umol/L; HC: head circumference; LT: liver transplantation; MM: medical management; NS: No significant. * In patient with LT is the amount of HAE/year before the LT. In patients on MM, it is the amount of HAE/year during follow-up.

## Data Availability

Data supporting the findings of this study are available from the corresponding author upon reasonable request due to legal and ethical reasons.

## Abbreviations

The following abbreviations are used in this manuscript:

ARG1D	Arginase 1 deficiency
ASLD	Argininosuccinate lyase deficiency
ASS1D	Argininosuccinate synthetase deficiency
CAVAD	Carbonic anhydrase VA deficiency
CITD	Citrin deficiency
CPS1D	Carbamoylphosphate synthetase 1 deficiency
EO	Early-onset
GPB	Glycerol phenylbutyrate
HAE	Hyperammonemia episode
HHH	Hyperornithinemia-hyperammonemia-homocitrullinuria syndrome
HRFS	High-risk family screening
INE	Instituto Nacional de Estadística
IQR	Interquartile range
LO	Late-onset
LT	Liver transplantation
MM	Medical management
NAGS	N-acetylglutamate synthase deficiency
NBS	Newborn screening
NaBZ	Sodium benzoate
NaPB	Sodium phenylbutyrate
ORNT1D	Ornithine/citrulline antiporter deficiency
OTCD	Ornithine transcarbamylase deficiency
REDcap	Research Electronic Data Capture
SD	Standard deviation
UCD	Urea cycle disorder
UCDC	Urea Cycle Disorders Consortium
